# Association between serum cotinine and learning disability in children aged 4–15 years: A secondary data analysis from the NHANES dataset

**DOI:** 10.18332/tid/205840

**Published:** 2025-07-23

**Authors:** Baomei He, Shengli Hu, Jingjing Jin, Yuanyuan Dai

**Affiliations:** 1 Center for Reproductive Medicine, Department of Pediatrics, Zhejiang Provincial People’s Hospital (Affiliated People’s Hospital), Hangzhou Medical College, Hangzhou, China; 2 Department of Stomatology, Hangzhou Linping District Hospital of Integrated Traditional Chinese and Western Medicine, Hangzhou, China; 3 Department of Pediatrics, Taizhou Central Hospital (Taizhou University Hospital), Taizhou, China

**Keywords:** serum cotinine, learning disability, secondhand smoke, National Health and Nutrition Examination Survey, children

## Abstract

**INTRODUCTION:**

While prior studies suggest links between secondhand smoke (SHS) exposure and developmental impairment, evidence linking objective biomarkers of SHS exposure to learning disability (LD) in children remains limited. This study investigates the association between serum cotinine – a validated biomarker of SHS exposure – and the higher likelihood of LD in US children.

**METHODS:**

This secondary analysis utilized cross-sectional data from the National Health and Nutrition Examination Survey (NHANES) 1999–2002, including 2573 children aged 4–15 years. Multivariable logistic regression models were implemented to evaluate the association between serum cotinine and parent-reported LD diagnoses. The dose-dependent relationship between cotinine and LD was analyzed using smooth curve fitting. Subgroup analyses were evaluated to assess robustness.

**RESULTS:**

Multivariable logistic regression analysis revealed that each unit increase in log-transformed cotinine was associated with a 1.81-fold increase in the odds of LD (AOR=1.81; 95% CI: 1.21–2.70, p<0.01). Children in the highest cotinine quartile exhibited 2.38-fold higher odds of LD compared to those in the lowest quartile (AOR=2.38; 95% CI: 1.23–4.58, p=0.01). Dose-response analysis revealed a linear relationship between log cotinine and LD (p for nonlinearity=0.20). Subgroup analyses further confirmed the stability of these results (p for interaction >0.05).

**CONCLUSIONS:**

The findings indicate a significant association between serum cotinine and an increased likelihood of LD in US children. The dose-dependent and linear nature of this relationship advocate for stricter smoke-free policies and targeted educational campaigns to reduce potential neurodevelopmental harms in children.

## INTRODUCTION

Learning disability (LD), characterized by difficulties in reading, writing, reasoning, or mathematical skills, affects approximately 5–15% of school-aged children worldwide, posing significant challenges to academic achievement and psychosocial well-being^1^. While genetic and perinatal factors are well-established contributors, emerging evidence suggests that environmental neurotoxicants, such as tobacco smoke, may disrupt neurodevelopmental processes^2-4^. Secondhand smoke (SHS) exposure, a modifiable risk factor, contains >7000 chemicals, including nicotine, which crosses the blood-brain barrier and may interfere with synaptic plasticity, neurotransmitter regulation, and cortical development^5,6^. Children are one of the most highly exposed populations^7^. Cross-country survey data show that the estimated national prevalence of household exposure to SHS among children aged >15 years ranged from 4.5% in Panama to 79.0% in Indonesia^8^. Due to their size- and age-specific behaviors and activity patterns, they are particularly vulnerable to cumulative SHS exposure and its related effects^8,9^. Despite this, studies investigating tobacco-specific biomarkers and LD remain limited, particularly in pediatric populations where critical neurodevelopmental windows heighten susceptibility to environmental exposures.

Cotinine, the primary metabolite of nicotine, serves as a validated biomarker for quantifying tobacco smoke exposure, and offers advantages over self-reported data by minimizing misclassification and recall bias^10-14^. Although prior studies have linked prenatal or childhood SHS exposure to cognitive deficits, attention problems, and behavioral disorders, findings on LD risk are inconsistent^15-18^. For instance, studies report mixed associations between cotinine levels and specific LD subtypes, potentially due to heterogeneous diagnostic criteria, small sample sizes, or inadequate adjustment for confounders such as socioeconomic status, lead exposure, or maternal education level^19^. Furthermore, few studies have explored dose-response relationships or stratified analyses, despite evidence suggesting that neurotoxic effects of nicotine may vary across developmental stages^15^.

To address these gaps, this study analyzed data from the National Health and Nutrition Examination Survey (NHANES), a nationally representative dataset with rigorous biospecimen collection and standardized cognitive assessments. By analyzing serum cotinine levels in children aged 4–15 years, we aim to: 1) evaluate the association between SHS exposure and parent-reported LD diagnoses; 2) assess potential influences of age, sex, and socioeconomic factors; and 3) quantify the dose-response relationship.

## METHODS

### Research design and data acquisition

**Figure 1:**
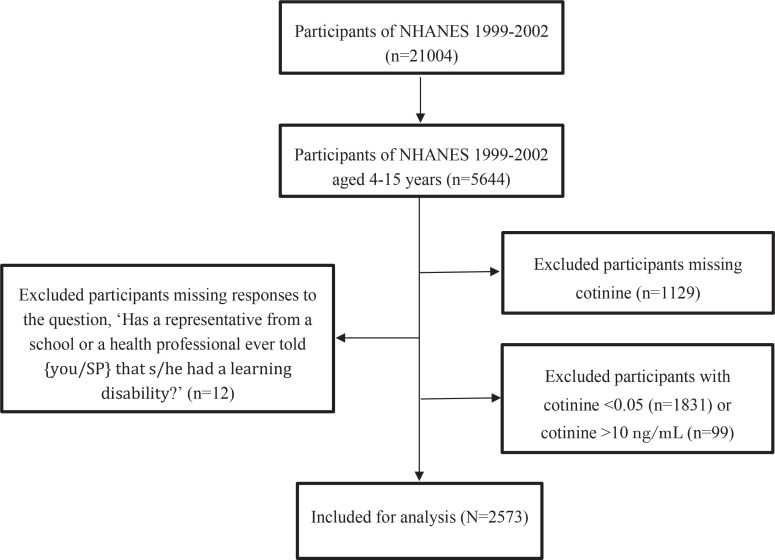
Flow chart of participant selection

The present secondary dataset analysis employed data from the 1999–2002 National Health and Nutrition Examination Survey (NHANES), a nationally representative survey of the US civilian non-institutionalized population conducted by the Centers for Disease Control and Prevention (CDC)^20^. The survey, initiated in the early 1960s and conducted annually since 1999, samples approximately 5000 participants from diverse geographical regions. The study period was selected because these survey cycles included assessments of LD in children aged 4–15 years. This study included a total of 2573 participants from the NHANES conducted between 1999 and 2002. Initially, 21004 participants were identified. Among these, a subset of 5644 individuals aged 4–15 years were surveyed with questions about LD. We then excluded: 1) 12 participants with missing responses to the LD diagnostic question, ‘Has a representative from a school or a health professional ever told you/your spouse that she/he had a learning disability?’; 2) 1831 participants with serum cotinine levels below the detection threshold (0.05 ng/mL); 3) 1129 participants with missing cotinine values; and 4) 99 participants with cotinine levels >10 ng/mL (indicative of active smoking). These exclusions yielded a final analytical sample of 2573 participants. Figure 1 presents a flow chart of the study participants, illustrating the inclusion and exclusion criteria.

### Assessment of serum cotinine and LD outcomes

Serum cotinine was measured by an isotope dilution-high performance liquid chromatography/atmospheric pressure chemical ionization tandem mass spectrometry. A child was considered to have cotinine-measured exposure if they had a detectable serum cotinine level of ≥0.05 ng/mL, consistent with previous analyses^7,21,22^. LD was defined based on parental or guardian reports of their child’s LD diagnosis.

### Variables

Covariates including age, sex, race/ethnicity, birth weight, blood lead levels, family PIR (poverty income ratio), parental education level, NICU admission status, maternal age at delivery, and daycare/preschool attendance were accounted for in the analysis. Covariate categorization followed NHANES protocols^20^. Race/ethnicity was self-reported and categorized into four groups: Mexican American, non-Hispanic White, non-Hispanic Black, and Other. Birth weight was converted to grams for analytical consistency, with the variable classified as <2500 g (low birth weight) and ≥2500 g. Parental education level was classified as more than high school, high school, and less than high school. Family PIR was categorized as: ≥4, <4 to ≥2, <2 to ≥1, and <1. Blood lead levels (μmol/L) were divided into tertiles: tertile 1 (≤1.1), tertile 2 (>1.1 to ≤1.9), and tertile 3 (>1.9).

### Statistical analysis

Data analysis utilized R (http://www.r-project.org) and EmpowerStats (http://www.empowerstats.com) for statistical modeling and sensitivity testing. Sample weights were applied per NCHS guidelines to ensure national representativeness. The dependent variable was LD, and the independent variable was serum cotinine. Serum cotinine levels were log transformed to achieve normal distribution. Missing covariate values were represented using dummy variables. Variables based on previous studies were incorporated as potential confounders^23-25^. Three hierarchical logistic regression models were developed: Model 1 included no adjusted covariates; Model 2 was adjusted for age, sex, and race/ethnicity; and Model 3 included all covariates in Table 1. The dose-response relationship between cotinine levels and LD was evaluated using generalized additive model and smoothing curve fittings. Further subgroup analyses and interaction tests were carried out to identify potential risk factors that could influence the association between cotinine levels and LD. A p<0.05 (two-sided) was considered statistically significant.

**Table 1:** Baseline characteristics of the study participants by quartiles of log cotinine (ng/mL), NHANES 1999–2002 (survey-weighted data)*

*Characteristics*	*Quartile 1 (<0.11)* *(N=616)*	*Quartile 2 (≥0.11 to <0.31)* *(N=670)*	*Quartile 3 (≥0.31 to <0.92)* *(N=641)*	*Quartile 4 (≥0.92)* *(N=646)*	*p*
**Age** (years)	9.61 (9.22–10.01)	9.39 (8.93–9.85)	9.80 (9.38–10.22)	9.34 (9.06–9.62)	0.37
**Sex**					0.38
Male	49.16 (42.63–55.72)	53.26 (47.29–59.14)	54.79 (48.95–60.51)	48.90 (43.66–54.17)	
Female	50.84 (44.28–57.37)	46.74 (40.86–52.71)	45.21 (39.49–51.05)	51.10 (45.83–56.34)	
**Race/ethnicity**					<0.01
Mexican American	16.71 (11.82–23.09)	12.91 (8.73–18.68)	9.74 (7.06–13.29)	4.13 (2.64–6.42)	
Non-Hispanic White	52.95 (43.94–61.77)	51.58 (42.55 –60.50)	50.58 (43.16–57.98)	67.26 (58.49–74.97)	
Non-Hispanic Black	17.13 (12.14–23.63)	20.52 (15.73–26.31)	26.76 (19.92–34.92)	20.69 (14.66–28.37)	
Other	13.21 (8.17–20.65)	14.99 (9.11–23.69)	12.93 (7.50–21.37)	7.92 (4.29–14.17)	
**Maternal age** (years)					
>18	91.45 (87.46–94.25)	87.22 (83.22–90.38)	87.28 (83.31–90.42)	81.08 (70.92–88.27)	0.01
≤18	8.55 (5.75–12.54)	12.78 (9.62–16.78)	12.72 (9.58–16.69)	18.92 (11.73–29.08)	
**LD**					
Yes	7.41 (4.89–11.08)	10.09 (6.32–15.73)	16.31 (12.58–20.88)	18.35 (15.13–22.07)	<0.01
No	92.59 (88.92–95.11)	89.91 (84.27–93.68)	83.69 (79.12–87.42)	81.65 (77.93–84.87)	
**Low birth weight**					
Yes	4.91 (3.20–7.48)	8.00 (5.57–11.36)	9.16 (6.69–12.42)	14.05 (8.58–22.18)	<0.01
No	95.09 (92.52–96.80)	92.00 (88.64–94.43)	90.84 (87.58–93.31)	85.95 (77.82–91.42)	
**Parental education level**					
High school or lower	27.66 (21.90–34.26)	24.56 (19.44–30.51)	32.26 (26.70–38.37)	41.14 (35.61–46.91)	<0.01
High school	24.22 (18.44–31.13)	37.19 (30.51–44.41)	37.04 (30.18–44.46)	35.63 (30.07–41.60)	
High school or higher	48.12 (41.37–54.95)	38.25 (32.25–44.63)	30.70 (23.90–38.45)	23.23 (17.19–30.61)	
**NICU admission**					
Yes	8.90 (6.24–12.56)	13.43 (9.75–18.20)	12.76 (9.64–16.72)	15.08 (11.90–18.93)	0.07
No	91.10 (87.44–93.76)	86.57 (81.80–90.25)	87.24 (83.28–90.36)	84.92 (81.07–88.10)	
**Daycare/preschool attendance**					
Yes	71.41 (64.72–77.28)	70.43 (64.99–75.35)	72.09 (65.36–77.94)	68.95 (62.48–74.75)	0.85
No	28.59 (22.72–35.28)	29.57 (24.65–35.01)	27.91 (22.06–34.64)	31.05 (25.25–37.52)	
**Family PIR**					
≥4	21.46 (16.51–27.40)	14.78 (10.32–20.71)	6.52 (4.22–9.93)	4.45 (2.16–8.95)	<0.01
≥2 to <4	31.57 (24.29–39.87)	24.32 (19.23–30.27)	23.31 (18.26–29.25)	20.38 (14.97–27.12)	
≥1 to <2	24.89 (19.64–31.00)	33.44 (25.72–42.17)	31.59 (25.26–38.68)	32.84 (23.22–44.16)	
<1	22.08 (16.37–29.10)	27.46 (21.47–34.38)	38.58 (30.84–46.95)	42.32 (33.40–51.78)	
**Blood lead level** (μg/dL)	1.58 (1.46–1.69)	1.72 (1.59–1.86)	1.98 (1.79–2.16)	2.12 (1.92–2.31)	<0.01

* Data presented for continuous variables are survey-weighted mean (95% CI); p-values were by survey-weighted linear regression. Data presented for categorical variables are survey-weighted percentage (95% CI); p-values were by survey-weighted chi-squared test. PIR: poverty income ratio. LD: learning disability. NICU: neonatal intensive care unit.

## RESULTS

### Baseline characteristics

The demographic and clinical characteristics of the participants, stratified by quartiles (Q1–Q4) of log-transformed cotinine levels, are presented in Table 1. The prevalence of LD rose significantly across quartiles, from 7.41% in Q1 to 18.35% in Q4 (p<0.01). Compared with children in the lower cotinine groups, those in the highest quartile (Q4) were more likely to have low birth weight, lower parental education level and family income, a higher proportion of maternal age ≤18 years at delivery, and elevated blood lead levels. The proportion of participants admitted to the NICU exhibited a borderline significant upward trend across quartiles, from 8.90% in Q1 to 15.08% in Q4 (p=0.07).

### Association between cotinine and LD

The association between cotinine levels and LD was examined using logistic regression models (Table 2). In the unadjusted model (Model 1), each unit increase in log cotinine was associated with a 2.00-fold increase in the odds of LD (95% CI: 1.59–2.52, p<0.01). After adjusting for age, sex, and race/ethnicity (Model 2), the adjusted odds ratio (AOR) increased to 2.09 (95% CI: 1.58–2.77, p<0.01). Further adjustment for birth weight, parental education level, NICU admission, daycare or preschool attendance, family PIR, maternal age at delivery, and blood lead level (Model 3) yielded an AOR of 1.81 (95% CI: 1.21–2.70, p<0.01). When serum cotinine levels were analyzed by quartiles, children in the highest quartile (Q4) exhibited significantly higher odds of LD compared to those in the lowest quartile (Q1), with an AOR of 2.38 (95% CI: 1.23–4.58, p=0.01) in the fully adjusted model.

**Table 2:** Multivariate analysis by quartiles of log cotinine logistic regression model, NHANES 1999–2002, (survey-weighted data)

*Variables*	*Model 1* *OR (95% CI) p*	*Model 2* *AOR (95% CI) p*	*Model 3* *AOR (95% CI) p*
Log cotinine (ng/mL)	**2.00 (1.59–2.52) <0.01**	**2.09 (1.58–2.77) <0.01**	**1.81 (1.21–2.70) <0.01**
Q1 (<0.11) ®	1	1	1
Q2 (≥0.11 to <0.31)	1.40 (0.72–2.75) 0.33	1.42 (0.72–2.82) 0.33	1.38 (0.51–3.72) 0.40
Q3 (≥0.31 to <0.92)	**2.43 (1.54–3.84) <0.01**	**2.34 (1.47–3.73) <0.01**	**2.16 (1.15–4.08) 0.02**
Q4 (≥0.92)	**2.81 (1.91–4.12) <0.01**	**2.91 (1.92–4.41) <0.01**	**2.38 (1.23–4.58) 0.01**
p for trend	**<0.01**	**<0.01**	**<0.01**

Model 1: no covariates were adjusted. AOR: adjusted odds ratio. Model 2: adjusted for age, sex, and race/ethnicity. Model 3: adjusted as for Model 2 plus birth weight, parental education level, NICU admission, daycare or preschool attendance, family poverty income ratio, maternal age at delivery, and blood lead level. The p-values for trend were calculated by treating log cotinine quartiles (Q1–Q4) as an ordinal variable. ® Reference category.

### Dose-response relationship between cotinine and LD

**Figure 2:**
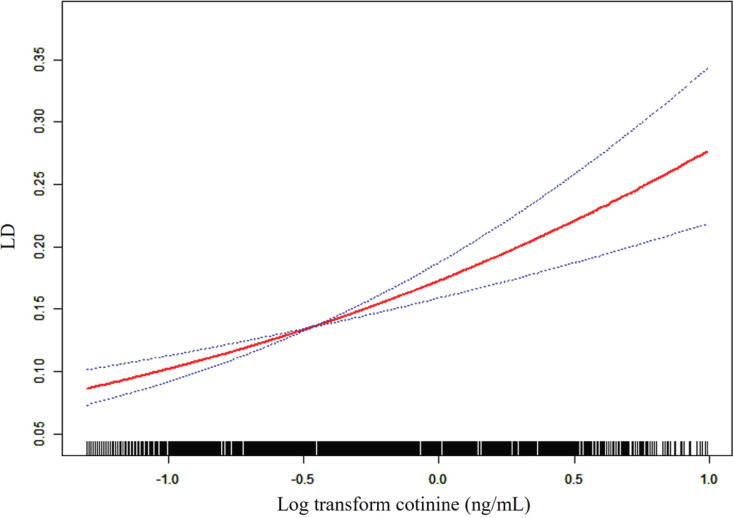
Relationship between cotinine and LD. The red line represents the relationship between log-transformed cotinine and LD, while the blue line represents the 95% confidence interval. Age, sex, race/ethnicity, birth weight, parental education level, NICU admission, day care or preschool attendance, family PIR, maternal age at delivery, blood lead level, were adjusted

Smooth curve fitting was used to assess the dose-response relationship between cotinine and LD. Multivariable-adjusted smooth curve fitting revealed a linear association between log cotinine and LD (p for nonlinearity = 0.20; Figure 2).

### Subgroup analysis

Subgroup analyses explored the association between serum cotinine and LD across demographic and clinical factors (Table 3). Participants aged 12–15 years exhibited an AOR of 2.10 (95% CI: 1.48–2.97, p=0.01). Males had an AOR of 1.71 (95% CI: 1.15–2.53, p=0.01), and females showed an AOR of 1.83 (95% CI: 1.10–3.03, p=0.05). Regarding race/ethnicity, Mexican Americans participants demonstrated the highest AOR (OR=3.05; 95% CI: 1.71–5.43, p=0.03). Low birth weight (<2500 g) was associated with an elevated AOR of 5.25 (95% CI: 1.19–23.11, p=0.03). Children with parents who had less than a high school education had an AOR of 2.14 (95% CI: 1.20–3.81, p=0.01). NICU admission was linked to an AOR of 3.38 (95% CI: 0.85–13.43, p=0.07), whereas non-attendance at daycare/preschool corresponded to an AOR of 2.17 (95% CI: 1.49–3.17, p<0.01). Maternal age ≤18 years at delivery was associated with an AOR of 2.51 (95% CI: 1.18–5.33, p=0.02). A family PIR <2 yielded AORs of 2.09 (<2 to ≥1) and 2.06 (<1). The highest tertile of blood lead levels (tertile 3) showed an AOR of 2.24 (95% CI: 1.33–3.78, p=0.04). The interaction tests revealed no significant effect modification across all stratified subgroups (p for interaction >0.05).

**Table 3:** Stratified logistic regression analysis of the association between cotinine and LD according to subgroup, NHANES 1999–2002 (survey-weighted data)

*Subgroups*	*Total* *n*	*AOR (95%CI)*	*p*	*p for interaction*
**Age** (years)				0.60
4–7	673	1.58 (0.78–3.22)	0.17	
8–11	759	1.90 (0.98–3.69)	0.05	
12–15	1141	2.10 (1.48–2.97)	0.01	
**Sex**				0.85
Male	1267	1.71 (1.15–2.53)	0.01	
Female	1306	1.83 (1.10–3.03)	0.05	
**Race/ethnicity**				0.24
Mexican American	695	3.05 (1.71–5.43)	0.03	
Non-Hispanic White	573	2.20 (1.18–4.11)	0.02	
Non-Hispanic Black	1092	1.55 (0.93–2.58)	0.08	
Other	213	0.92 (0.34–2.46)	0.87	
**Low birth weight**				0.19
Yes	254	5.25 (1.19–23.11)	0.03	
No	2190	1.64 (1.08–2.50)	0.02	
**Parental education level**				0.36
High school or lower	1091	2.14 (1.20–3.81)	0.01	
High school	700	1.98 (0.84–4.64)	0.15	
High school or higher	703	1.99 (1.08–3.65)	0.03	
**NICU admission**				0.43
Yes	284	3.38 (0.85–13.43)	0.07	
No	2267	1.78 (1.29–2.45)	0.01	
**Daycare/preschool attendance**				0.80
Yes	1710	1.56 (1.10–2.20)	0.03	
No	861	2.17 (1.49–3.17)	<0.01	
**Maternal age** (years)				0.87
≤18	409	2.51 (1.18–5.33)	0.02	
>18	2129	1.81 (1.21–2.69)	<0.01	
**Family PIR**				
≥4	193	2.47 (0.19–32.00)	0.40	0.84
≥2 to <4	498	1.38 (0.64–2.95)	0.37	
≥1 to <2	756	2.09 (1.15–3.80)	0.02	
<1	1126	2.06 (1.33–3.16)	<0.01	
**Blood lead level** (μmol/L)				0.52
Tertile 1 (≤1.1)	820	1.45 (0.87–2.42)	0.23	
Tertile 2 (<1.1 to ≤1.9)	844	1.84 (1.14–2.96)	0.05	
Tertile 3 (>1.9)	907	2.24 (1.33–3.78)	0.04	

AOR: adjusted odds ratio; adjusted for age, sex, race/ethnicity, birth weight, parental education level, NICU admission, daycare or preschool attendance, family poverty income ratio (PIR), maternal age at delivery, blood lead level, excluding the stratification variable.

## DISCUSSION

The findings of this cross-sectional study utilizing NHANES data demonstrate a significant positive association between serum cotinine – a biomarker of secondhand smoke (SHS) exposure – and an increased likelihood of learning disability (LD) in US children aged 4–15 years. After full adjustment for covariates, every 1-unit increase in log-transformed cotinine was associated with a 1.81-fold increase in the odds of LD. Children in the highest cotinine quartile exhibited 2.38-fold higher odds of LD compared to those in the lowest quartile. Dose-response analysis revealed a linear relationship between log cotinine and LD (p for nonlinearity = 0.20). Subgroup analyses further confirmed the stability of these results.

Our results align with existing evidence linking SHS to adverse neurocognitive outcomes, though prior studies have predominantly focused on prenatal or direct maternal smoking^26-29^. The observed association between postnatal SHS exposure and LD underscores the potential vulnerability of school-aged children to environmental neurotoxicants. This relationship is biologically plausible, as experimental models demonstrate that nicotine and its metabolites disrupt synaptic plasticity, impair cholinergic signaling, and induce oxidative stress in developing neural circuits^29-32^.

Subgroup analyses revealed that the association between serum cotinine and LD strengthened progressively with age, suggesting a potential cumulative effect of exposure. These findings underscore the importance of early intervention; targeted strategies to identify and mitigate children’s exposure to hazardous substances could reduce the risk of LD development. The subgroup with a birth weight <2500 g exhibited the strongest association across different birth weight groups. This result supports previous research indicating that low birth weight may negatively impact neurodevelopment, thereby increasing susceptibility to LD^33^. Notably, our stratified analyses further revealed stronger associations in those with lower socioeconomic status, suggesting demographic subgroups that may require targeted interventions. Families with lower socioeconomic status are disproportionately exposed to household smoking and often face limited access to educational resources, creating a syndemic effect on neurodevelopment^34,35^.

### Strengths and limitations

Several methodological strengths enhance the validity of these findings. The use of serum cotinine – a quantitative, objective biomarker – minimizes misclassification and recall bias inherent in self-reported smoke exposure. The nationally representative NHANES sample ensures generalizability to the US pediatric population, while the inclusion of covariates such as lead levels, socioeconomic status and nutritional factors addresses key environmental confounders.

However, the study’s cross-sectional design limits causal interpretation, as the temporality between cotinine levels and LD diagnosis remains uncertain. Moreover, reverse causation, wherein children with LD are more likely to reside in environments with higher smoking rates, cannot be ruled out. Additionally, the binary classification of LD based on caregiver reports (‘Has a representative from a school or a health professional ever told you/your spouse that she/he had a learning disability?’) may lack the precision of clinical or academic assessments, potentially underestimating subtle cognitive deficits. Furthermore, despite adjusting for key covariates, residual confounding may persist due to unmeasured factors, such as dietary habits, environmental pollutants, or genetic predispositions. Finally, since the study population comprised only US children, the findings may not generalize to other countries with differing smoking prevalence, cultural practices, or healthcare systems.

## CONCLUSIONS

This study demonstrates that serum cotinine is independently associated with LD in school-aged children. The results reinforce the need for pediatricians to screen for tobacco smoke exposure during developmental assessments and for policymakers to prioritize SHS reduction as a modifiable determinant of educational inequities. Mitigating environmental neurotoxicant exposure may serve as a critical strategy for improving neurodevelopmental outcomes in vulnerable pediatric cohorts.

## Data Availability

The data supporting this research are available from the following source: http://cdc.gov/nchs/nhanes
